# Regenerative, Highly-Sensitive, Non-Enzymatic Dopamine Sensor and Impact of Different Buffer Systems in Dopamine Sensing

**DOI:** 10.3390/bios8010009

**Published:** 2018-01-24

**Authors:** Saumya Joshi, Vijay Deep Bhatt, Andreas Märtl, Markus Becherer, Paolo Lugli

**Affiliations:** 1 Department of Electrical Engineering and Information Technology, Institute for Nanoelectronics, Technische Universität München, 80333 Munich, Germany; vijay.bhatt@tum.de (V.D.B.); ga34qen@mytum.de (A.M.); markus.becherer@tum.de (M.B.); 2 Faculty of Science and Technology, Free University of Bozen-Bolzano, 39100 Bolzano, Italy; Paolo.Lugli@unibz.it

**Keywords:** carbon nanotube, non-enzymatic, dopamine sensors, buffer

## Abstract

Carbon nanotube field-effect transistors are used extensively in ultra-sensitive biomolecule sensing applications. Along with high sensitivity, the possibility of regeneration is highly desired in bio-sensors. An important constituent of such bio-sensing systems is the buffer used to maintain pH and provide an ionic conducting medium, among its other properties. In this work, we demonstrate highly-sensitive regenerative dopamine sensors and the impact of varying buffer composition and type on the electrolyte gated field effect sensors. The role of the buffer system is an often ignored condition in the electrical characterization of sensors. Non-enzymatic dopamine sensors are fabricated and regenerated in hydrochloric acid (HCl) solution. The sensors are finally measured against four different buffer solutions. The impact of the nature and chemical structure of buffer molecules on the dopamine sensors is shown, and the appropriate buffer systems are demonstrated.

## 1. Introduction

Dopamine is a chemical from the catecholamine and phenethylamine families, and performs crucial functions in the brain and the central nervous system (CNS) as a neurotransmitter and as a local chemical messenger that controls the immune system and digestive system, among others [1,2,3]. Dysfunctions in dopamine systems can lead to serious medical and neurological conditions, such as Parkinson’s disease [4,5], Alzheimer’s disease [6], depression, addiction, and schizophrenia. Analytical methodologies that can be used for dopamine concentration measurements include high-performance liquid chromatography [7], capillary electrophoresis [8], mass spectroscopy [9], and electrochemistry [10]. However, these techniques cannot provide real-time and rapid dopamine detection, which can open the possibility for the loco-regional therapy of neurodegenerative diseases [11]. To achieve this goal, several demonstrations have been made for biosensors based on field-effect transistors (FETs) [12,13,14]. Interest in FET-based biosensors is fomented by their desirable characteristics, such as rapid label-free electrical detection, low power consumption, portability, inexpensive mass production, and the possibility of on-chip integration of both sensor and measurement systems [15].

Carbon nanotube field effect transistor (CNTFET)-based biosensors have attracted a great deal of attention due to their unique electronic properties, special geometry (high surface area-to-volume ratio), high mechanical strength, and chemical stability [16,17]. In this work we report a highly-sensitive dopamine sensor with the lowest concentration that can be resolved in a range as low as fM. The selectivity is achieved by modifying the flexible substrate using a non-enzymatic scheme [18]. Although non-enzymatic sensors overcome the drawbacks of enzymatic sensors (e.g., high cost, complicated production procedures, and short shelf lives) to a large extent, they still suffer from challenges like stability and selectivity [19]. In the case of dopamine sensors, achieving complete selectivity is not straightforward, as dopamine coexists with various electroactive molecules [20]. However, the ability to have high sensitivity up to the fM range makes this sensor highly lucrative compared to other recently demonstrated FET-based dopamine sensors—for example, by Park et al. where dopamine receptors were used in nanohybrid [1,20] and measured lowest concentrations in the nM range, by Lee et al. using modified platinum nanoparticles where the lowest measured concentration was 100 fM [21], by Zhang et al. using graphene as semiconductor with reported lowest concentration in the nM range [22].

In addition to the highly sensitive experimental conditions like the effect of counterions, ionic strength of buffer also plays a role in deciding the performance of the biosensor [23]. Researchers have demonstrated on silicon nanowire FETs that such conditions can affect the Debye screening and influence the response of the sensor [24,25]. Debye length, a characteristic of Debye screening, is the distance at which a unit charge is reduced to 1/e ≈ 0.37, meaning that the protein charges are screened by 63% [26]. We observed that along with the effect of ionic strength and pH of the underlying buffer, the buffer composition has a severe impact on the response of the sensor. As a part of this work, we have challenged the dopamine CNTFET sensors in different buffer solutions, and large differences were seen in the sensor’s response with varying buffer solution. The measurements were done at almost the pKa value for each buffer to ensure the optimal buffering capacity [27].

Another big challenges in the commercial biosensor market is the cost of the sensors. Although expensive sensors are used extensively in research environments, low cost sensors have higher market potential. The high cost in sensor development is mainly attributed to high cost of instrumentation and materials. In addition to printed technologies and ambient processing techniques which are already leading to economical sensor development, the possibility of reusable sensors can be another way to further reduce the costs. Apart from economic reasons, the reusability of sensors is inevitable in applications where device-to-device variance can be a major source of error. Hence, the regeneration of sensors is of high interest among researchers [28]. The final part of this work presents a demonstration of the regeneration of the dopamine sensor by acidic treatment.

## 2. Materials and Methods

### 2.1. Fabrication of Flexible CNTFET

**Figure 1:**
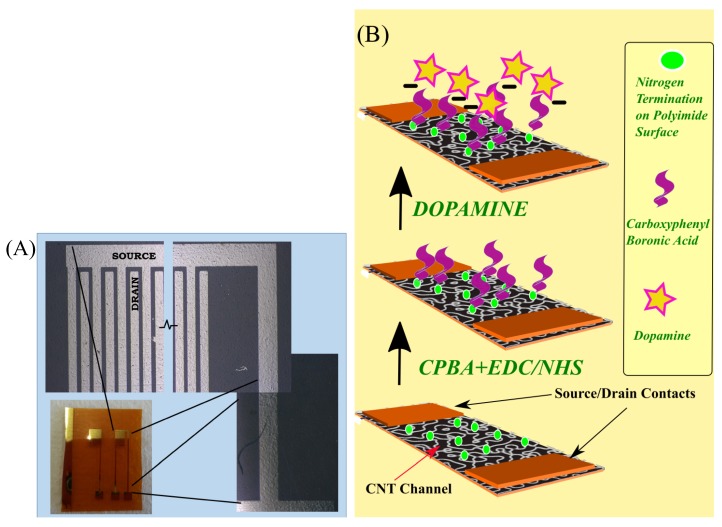
(**A**) Picture and optical microscope images showing the interdigitated electrodes (IDEs) and contact lines of the flexible carbon nanotube field effect transistor (CNTFET); (**B**) Schematic representation of the functionalization scheme. CBPA: carboxyphenyl boronic acid; EDC: *N*-ethyl-*N*${}^{\prime }$- (3-dimethylamino-propyl) carbodiimide hydrochloride; NHS: *N*-hydroxysuccinimide.

Patterns of source, drain, and gate contacts were formed by standard negative photo-lithography followed by lift-off process. On the patterned flexible polyimide substrate (Kapton, DuPont, 300 HN, 150 μm) 5 nm-thick Cr (adhesion promoter layer) and 40 nm Au were thermally evaporated as contact metals. The active channel area of IDEs (interdigitated electrodes) structure had an aspect ratio (channel width/length) equal to 900 and the channel length was 50 μm. Figure 1A shows a picture of the sensor, with a zoomed-in image showing the optical microscope images over the IDEs and the contact lines. For the active channel, semiconducting carbon nanotubes (CNTs) were dispersed in the aqueous medium surfactant sodium dodecyl sulfate (SDS) [29], and this CNT solution was sprayed over the structures using a shadow mask. The automated spray system used for spraying was equipped with an industrial air atomizing spray valve (Nordson EFD, East Providence, RI, USA) in combination with an overhead motion platform (Precision Valve & Automation, Cohoes, NY, USA). More details about the spray deposition process set-up can be found elsewhere [29,30]. The samples were immersed in DI-H${}_{2}$O for 15 min at room temperature to remove the remaining surfactant, and the devices were subsequently dried with nitrogen.

### 2.2. Functionalization of the CNTFETs for Dopamine Sensing

After device fabrication, the flexible CNTFETs must be functionalized such that they are selective to dopamine. Carboxyphenyl boronic acid (CPBA) has the ability to bind with dopamine and form a charged boronate ester which can then modulate the surface charge on the semiconducting channel [12,31,32]. Traditionally, the first step of such a functionalization scheme is to introduce an amine group on the substrate, usually achieved by immersing the substrate in 3-aminopropyltriethoxysilane (APTES) [31,33]. However, working with the polyimide substrate provides the advantage of eliminating this step as the imide groups on the polyimide surface offer the opportunity to bind CPBA directly without an extra linking molecule [18].

The final functionalization scheme used for sensors in this study is illustrated in Figure 1B. EDC (*N*-Ethyl-*N*${}^{\prime }$-(3-dimethylamino-propyl) carbodiimide hydrochloride)/NHS (*N*-hydroxysuccinimide) chemistry for the activation of carboxylic acids was exploited [34]. For this, 30 μM EDC, 30 μM CPBA, and 30 μM NHS were prepared separately in 1 mM MES (2-(*N*-morpholino)ethanesulfonic acid). Equal amounts (20 μL) each of these three solutions were dropcasted in succession on the active area of the CNTFET. The samples were kept at the ambient atmosphere. Once all the liquid evaporated (normally after 3–4 h), the samples were thoroughly rinsed with MES buffer and air dried.

**Figure 2:**
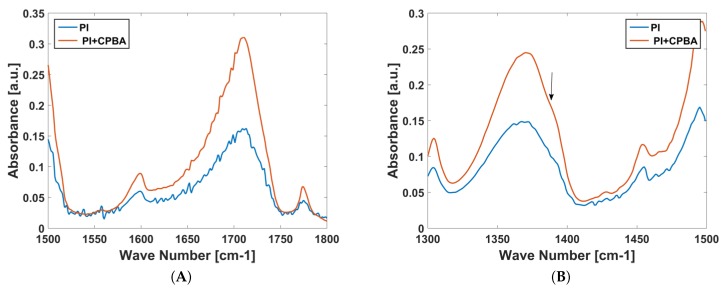
Attenuated total reflection Fourier transform infrared spectroscopy (ATR-FTIR) spectra of polyimide (PI) film before and after functionalization with CPBA in the range (**A**) 1500 cm${}^{-1}$ to 1800 cm${}^{-1}$; (**B**) 1300 cm${}^{-1}$ to 1500 cm${}^{-1}$.

Fourier transform infrared (FTIR) measurements were performed on the kapton sample before and after it was functionalized with CPBA using the Alpha spectrometer (Bruker Optics GmbH, Ettlingen, Germany) controlled by OPUS software in the attenuated total reflection (ATR) mode. The spectral resolution was chosen as 2 cm${}^{-1}$, and 24 scans were recorded and averaged per sample. Figure 2A shows the spectra of bare polyimide (PI) and the polyimide functionalized with CPBA (PI+CPBA) zoomed-in on the range from 1500 cm${}^{-1}$ to 1800 cm${}^{-1}$. The width and height of the C=O peaks around 1700 were modified after functionalization, indicating a change in the environment [35]. The second observation is the suppression of the small N-O peaks slightly below 1550 cm${}^{-1}$ after the polyimide was functionalized with CPBA. Figure 2B shows the spectra in the range 1300 cm${}^{-1}$ to 1500 cm${}^{-1}$; a slight peak seen just above 1380 cm${}^{-1}$ is a signature of the B-O bond of the boronic acid [36], hence confirming successful surface functionalization.

### 2.3. CNTFET Electrical Characterization

**Figure 3:**
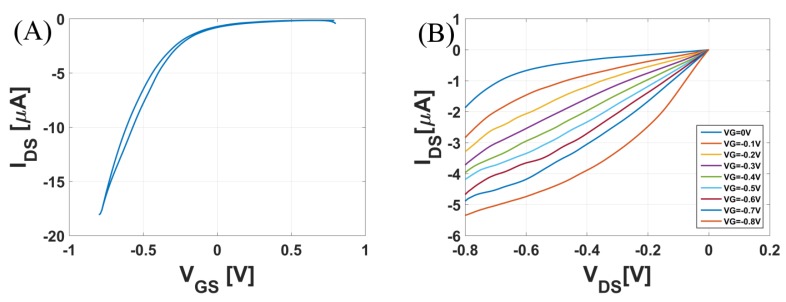
For an un-functionalized CNTFET: (**A**) Transfer Curve where V${}_{GS}$ varies from 0.8 V to −0.8 V and V${}_{DS}=-0.1$ V. (**B**) Output Curve with V${}_{DS}$ varying from 0 to −0.8 V for different values of V${}_{GS}$.

The electrical characterizations of the CNTFETs was performed at ambient conditions using a Keithley Source Measuring Unit (SMU). For all electrical measurements, a PDMS chamber was mounted around the active area of the CNTFETs to serve as a compartment for around 60 μL of the analyte solution, which was exchanged manually using a Gilson pipette. Figure 3A,B are the transfer curves and output curves for the un-functionalized flexible CNTFETs. Important transistor parameters were computed: the on-off ratio was 125, threshold voltage using the ELR (Extrapolation in the Linear Region) method was −0.52 V, and the maximum transconductance was 58.11 μS. The applied voltages were selected in the range below ±1 V to prevent any electrochemical reaction.

## 3. Results and Discussion

### 3.1. A Dopamine Sensor: Measured in Phosphate-Buffered Saline  (PBS) Solution

**Figure 4:**
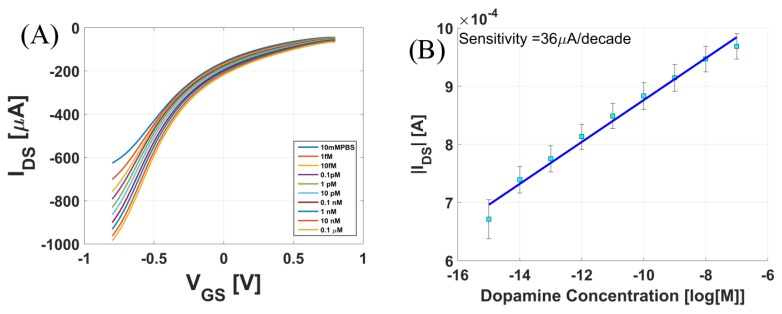
(**A**) Response of the sensor to varying concentrations of dopamine in 10 mM PBS (phosphate-buffered saline); (**B**) Maximum drain-to-source current vs. concentration of dopamine. Calculated sensitivity of the sensor is 36 μA/decade.

Figure 4A shows the transfer curves of functionalized CNTFET recorded with varying concentrations of dopamine in 10 mM PBS buffer solution at pH value chosen at its pKa (i.e., pH = 7). Figure 4B is the maximum current response as a function of the dopamine concentration for voltages V${}_{DS}=-0.1$ V and V${}_{GS}=-0.8$ V. Using a linear fit in this curve, we extracted the sensitivity of the sensor as 36 μA/decade. As seen from Figure 4A,B, as the concentration of dopamine increased from 1 fM to 0.1 μM, the drain-to-source current (I${}_{DS}$) of the transistor increased. Post-functionalization, the polyimide surface is activated with boronic acid. The dopamine mixed in the buffer solution binds to the boronic acid and forms boronate ester as illustrated in Figure 1B. The formed boronate ester has a negative charge on the boron atom and influences the device characteristics of the CNTFET. With the increase in dopamine concentration, there was an increase in the negative charge near the surface of the p-type semiconducting channel. This negative charge would attract more holes (majority carriers) on the surface of CNTs, thus causing an increase in the current for p-type FET.

### 3.2. Comparison with Other Buffers

**Figure 5:**
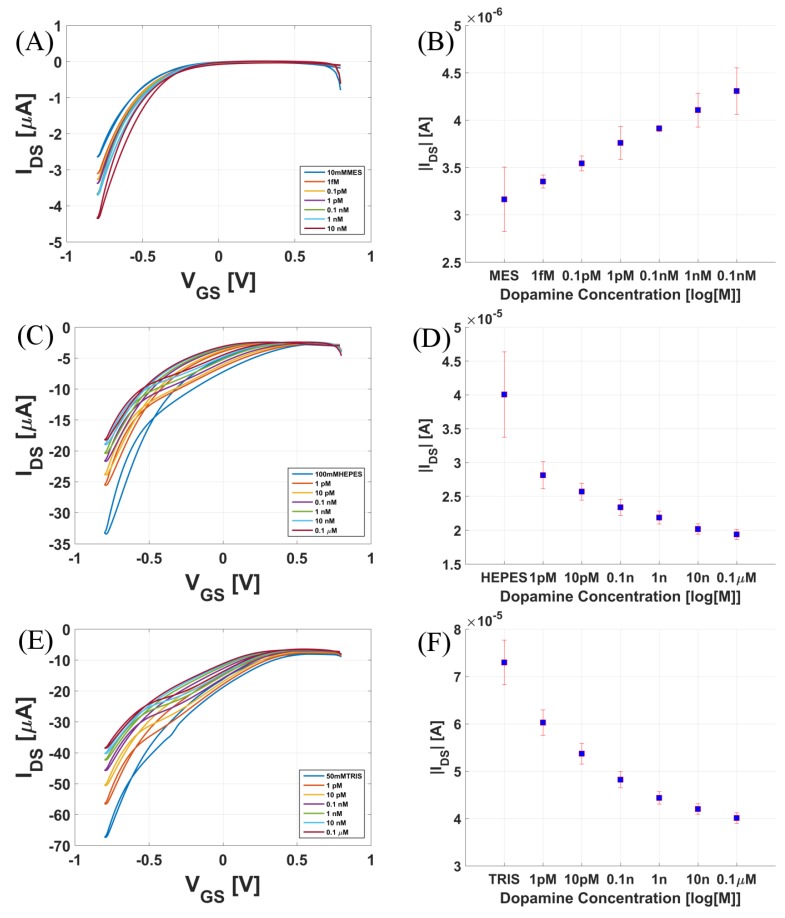
Transfer curves recorded for varying concentrations of dopamine in (**A**) 10 mM MES (2-(*N*-morpholino)ethanesulfonic acid; pH = 6.17); (**C**) 100 mM HEPES (pH = 7); (**E**) 50 mM TRIS buffer (pH = 8). Maximum I${}_{DS}$ vs. concentration of dopamine in (**B**) 10 mM MES (pH = 6.17); (**D**) 100 mM HEPES (pH = 7) (4-(2-Hydroxyethyl)piperazine-1-ethanesulfonic acid); (**F**) 50 mM TRIS buffer (pH = 8) (Tris(hydroxymethyl)aminomethane).

Figure 5 shows the response of different sensors to varying concentrations of dopamine in MES, HEPES (4-(2-Hydroxyethyl)piperazine-1-ethanesulfonic acid), and TRIS (tris(hydroxymethyl) aminomethane) buffers. Figure 5A,C,E are the transfer curves recorded in forward and backward sweep directions. Figure 5B,D,F are the corresponding maximum current vs. concentration curves. The results clearly indicate that as the concentration of dopamine increased there was an increase in drain current when dopamine solutions were prepared in MES buffer. This behavior is similar to that recorded with varying dopamine concentrations in PBS buffer (Figure 4A,B). However, when dopamine solutions were prepared in TRIS and HEPES buffer, we observed that the current decreased upon moving towards higher dopamine concentrations. This current vs. concentration trend is consistent when measured over several devices. Figure S1 shows the statistical data for the four buffer solutions under investigation. Each curve is averaged over five devices.

**Figure 6:**
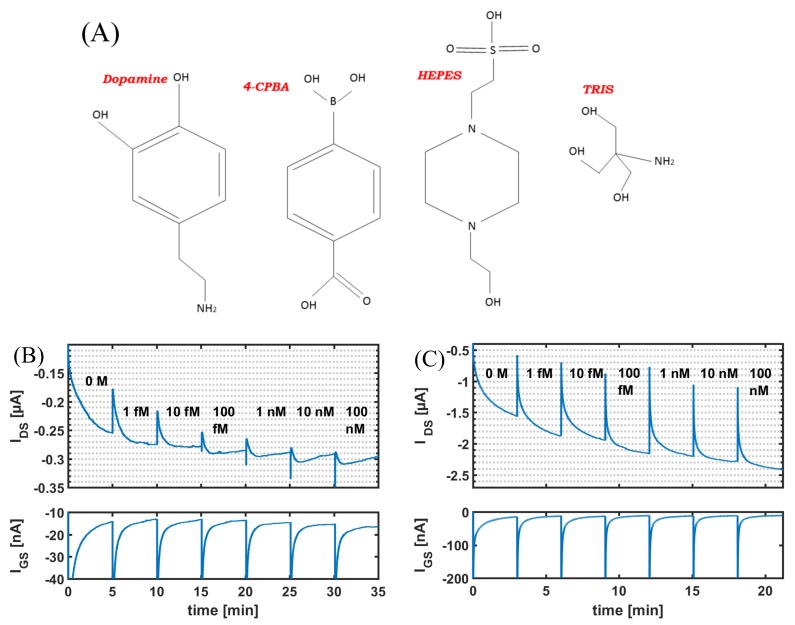
(**A**) Chemical Structure for dopamine, CPBA, TRIS, and HEPES. Real-time response of functionalized CNTFET to varying concentrations of (**B**) HEPES and (**C**) TRIS in 10 mM PBS with constant applied bias V${}_{DS}=-0.1$ V and V${}_{GS}=-0.8$ V.

To investigated the “unexpected” response of sensors to varying dopamine concentrations in HEPES and TRIS buffer, we look at the chemical structure of dopamine, TRIS, HEPES, and CPBA in Figure 6A. To form the boronate ester, three hydroxyl groups come together and a condensation reaction takes place. Looking at the chemical structure of HEPES and TRIS, it is clear that both the molecules have -OH groups such that an esterification reaction can also take place between these buffer molecules and CPBA, instead of CPBA and dopamine. To confirm this, we measured the response of the sensor to varying concentrations of TRIS and HEPES in 10 mM PBS buffer solution, as shown in Figure 6B,C. As the concentration of TRIS and HEPES increased there was an increase in the drain-to-source current. It is important to see that this is not a faradic current, as no change was seen in the gate-to-source current (I${}_{GS}$). These measurements clearly indicate that the HEPES and TRIS molecules interact with the functionalization (CPBA) and hence interfere with the normal operation of the sensor.

**Figure 7:**
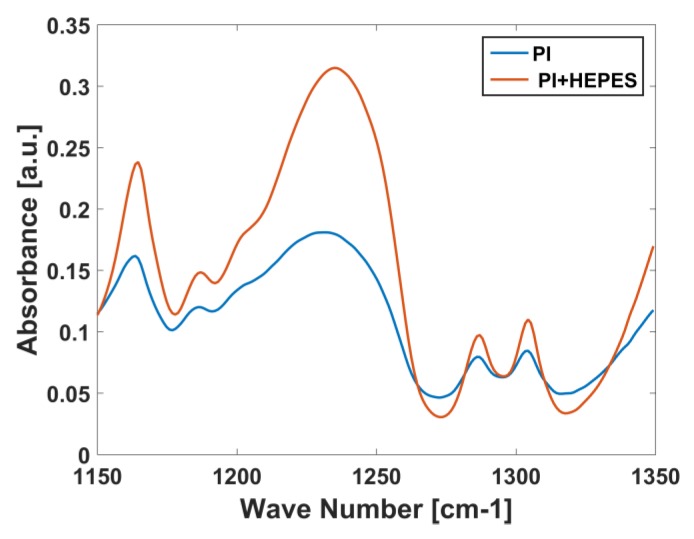
ATR -FTIR spectra of bare polyimide substrate (PI) and after a dopamine sensor on such a substrate is with HEPES solution (PI+HEPES).

THE ATR-FTIR spectra of sensors after they were measured in varying concentration of HEPES (measurement of Figure 6B) were also recorded. Figure 7 compares such spectra with that of a bare polyimide film. An increase in the band just below 1250 cm${}^{-1}$ is seen, which originates mainly from the O=S=O vibration [37] and hence indicates that HEPES molecules have a tendency to bind to the functionalization.

### 3.3. Regeneration Using Acidic Solution

The regeneration of sensors will assist in re-usability and increasing the commercial viability of sensors. It is observed that for the regeneration of biosensors, the solvent environment needs to be altered in such a way that the analyte/receptor binding is weakened. The main reagents used to achieve this are acid/base (pH change), detergents, glycine, and urea [28]. For dopamine regeneration, we have demonstrated use of HCl solution [38].

**Figure 8:**
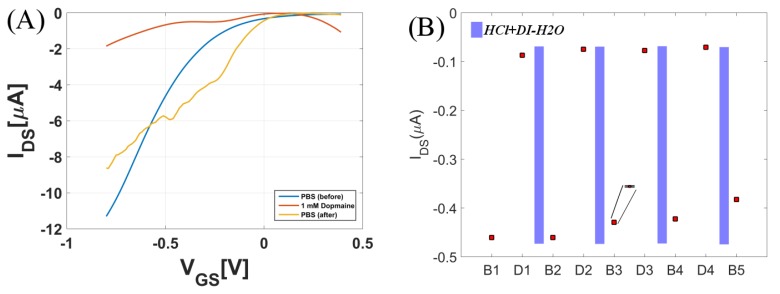
Regeneration of CNTFET dopamine sensor: (**A**) after treatment with 0.5 M HCl; (**B**) four regeneration cycles where B(1,2,3,4,5) refer to the number of times the sensor was measured in buffer and in between the 1mM dopamine measurements D(1,2,3,4), the blue bar indicates HCl treatment.

For very high concentrations of dopamine (∼1 mM), the sensor saturates and cannot be turned on, as seen in Figure 8A. This can be partly because the Debye length significantly decreases for very high concentrations of dopamine. After the measurement in highly concentrated dopamine solution, the CNTFET was rinsed thoroughly with 0.5 M hydrochloric acid and later throughly rinsed with DI-H${}_{2}$O. As seen from Figure 8A, the dopamine sensor recovered and the CNTFET turned on with a loss of about 20% in the maximum current after it was treated with HCl. In an attempt to recover the device more effectively, a lower concentration of HCl (10 mM) was used after the device was subjected to 1 mM dopamine. Figure 8B depicts the I${}_{DS}$ of the measurement where first the device was measured in buffer solution (B1), and then with dopamine (D1). After this, the device was exposed to 10 mM HCl for 5 min and subsequently thoroughly washed with DI-H${}_{2}$O. This process of regenerating the sensors with HCl after measuring in buffer B(1,2,3,4,5) and dopamine D(1,2,3,4) was performed four times. It was observed that there was a 19% decrease in the current level after four regeneration cycles with 10 mM HCl.

## 4. Conclusions

A flexible dopamine sensor using a CNT field effect transistor has been demonstrated. Measurements in PBS buffer indicate a highly-sensitive sensor. Measurements were also performed for three other buffer solutions, namely HEPES, MES, and TRIS. The results clearly indicate that along with the most widely considered parameters like pH and ionic strength of the buffer, the chemical nature of buffer must also be considered. It is demonstrated that HEPES and TRIS buffers can themselves react to the functionalization, and the measurements in these buffer systems no longer hold correct in measuring dopamine. Finally, the regeneration of this dopamine sensor by treatment with acidic solutions is demonstrated.

## Supplementary Materials

The following are available online at http://www.mdpi.com/2079-6374/8/1/9/s1, Figure S1: Statistical Analysis of dopamine sensors (each curve represents measurements of five different sensors) measured in (A) 10 mM PBS (pH = 7) (B) 10 mM MES (pH = 6.17) (C) 100 mM HEPES (pH = 7) (D) 50 mM TRIS buffer (pH = 8). (VDS = −0.1 V and VGS = −0.8).
