# Supplementary material for: Regenerative, Highly-Sensitive, Non-Enzymatic Dopamine Sensor and Impact of Different Buffer Systems in Dopamine Sensing
Source: Biosensors (Basel). 2018 Jan 24;8(1):9. doi: 10.3390/bios8010009 (PMC5872057; doi:10.3390/bios8010009)
Supplement: Supplementary file 1 [file biosensors-08-00009-s001.pdf]

Regenerative, highly-sensitive, non-enzymatic dopamine sensor and impact of different buffer systems in dopamine sensing

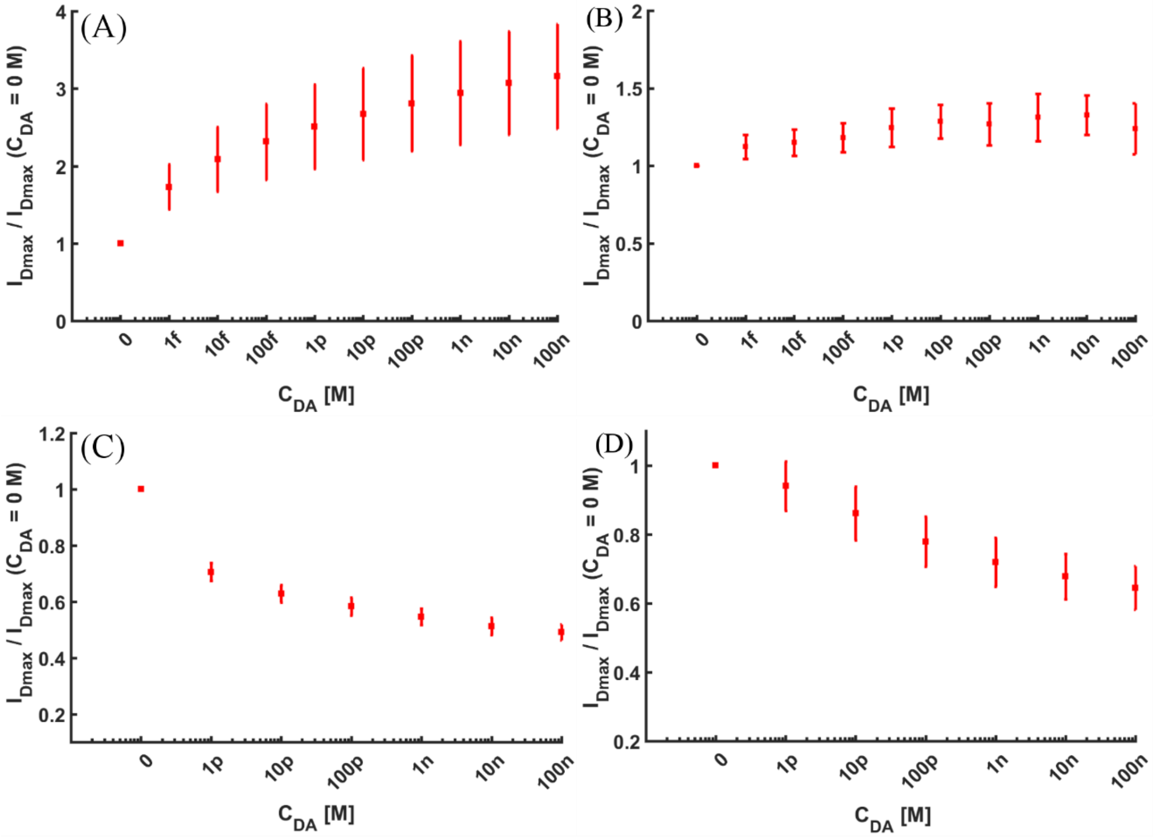

Figure S1: Statistical Analysis of dopamine sensors (each curve represents measurements of five different sensors) measured in (A) 10mM PBS(pH=7) (B) 10mM MES(pH=6.17) (C) 100mM HEPES(pH=7) (D) 50mM TRIS buffer(pH=8). (V_{DS}=-0.1V and V_{GS}=-0.8)
